# Hepatocyte growth factor and B-type natriuretic peptide as independent predictors of mortality in HFpEF patients

**DOI:** 10.3389/fcvm.2025.1512411

**Published:** 2025-02-18

**Authors:** Hou-liang Chen, Xue-tao Zhu, Wang Zhang, Xiao-bing Cheng, Ze-ping Hu

**Affiliations:** ^1^ Department of Cardiology, The First Affiliated Hospital, Anhui Medical University, Hefei, Anhui, China; ^2^ Department of Cardiology, The Third People's Hospital of Hefei (Hefei Third Clinical College, Anhui Medical University), Hefei, Anhui, China; ^3^ Department of Pharmacy, Hefei Third Clinical College, Anhui Medical University (Hefei Third People’s Hospital), Hefei, Anhui, China

**Keywords:** HGF, BNP, all-cause mortality, HFpEF, retrospective cohort study

## Abstract

**Background:**

Heart failure with preserved ejection fraction (HFpEF) is a common and heterogeneous syndrome with high mortality and morbidity. However, few studies have evaluated the relationship between biomarkers and subsequent outcomes in HFpEF patients.

**Objective:**

To assess the association between plasma hepatocyte growth factor (HGF) levels and all-cause mortality in HFpEF patients.

**Methods:**

This was a retrospective cohort study of 412 HFpEF patients who were hospitalized in the Department of Cardiology of the First Affiliated Hospital of Anhui Medical University from November 2020 to November 2021. The patients were divided into two groups according to the 24-month follow-up results: deceased (82 cases) and survivors (330 cases). The primary outcome was all-cause mortality. Multivariate logistic regression analysis was performed to identify the risk factors for all-cause mortality in HFpEF patients. Receiver operating characteristic (ROC) curve analysis was used to evaluate the predictive value of relevant indicators for HFpEF mortality risk. Kaplan–Meier analysis was used to assess the risk of all-cause mortality in patients with increased relevant indicators.

**Results:**

Multivariate logistic regression analysis showed that HGF, B-type natriuretic peptide precursor (BNP), total protein (TP), estimated glomerular filtration rate (eGFR), and tetraiodothyronine (T4) were independent risk factors for all-cause mortality in HFpEF patients (*P* < 0.05). ROC curve analysis showed that the optimal cut-off point of HGF was 1,598 pg/ml [area under the curve (AUC) = 0.645, *P* = 0.000, hazard ratio (HR) = 3.186, 95% confidence interval (CI): 1.963–5.171], the optimal cut-off point of BNP was 271 pg/ml (AUC = 0.703, *P* < 0.000, HR = 4.494, 95% CI: 2.914–6.930), and the optimal cut-off point of eGFR was 114.5 ml/min/1.73 m^2^ (AUC = 0.644, *P* = 0.423). Kaplan–Meier survival curve analysis showed that the survival probability of the patients with low HGF and BNP concentrations was significantly higher (*P* < 0.0001), while there was no significant difference in the survival rate between the two subgroups with eGFR as the cut-off value (*P* = 0.423).

**Conclusion:**

HGF and BNP are independent risk factors for all-cause mortality events in HFpEF patients during 24 months of follow-up, and the survival probability of HFpEF patients with low HGF and BNP concentrations is higher.

## 1 Introduction

Heart failure (HF) is a major cause of mortality and morbidity from cardiovascular diseases. Among HF patients, those with preserved ejection fraction (HFpEF) have a similar mortality rate as those with reduced ejection fraction (HFrEF), ranging from 53% to 74% in 5 years (1). HFpEF is a heterogeneous syndrome with multiple etiologies and pathophysiological mechanisms. However, the clinical manifestations of HFpEF are nonspecific and often overlap with other comorbidities, making the diagnosis and treatment of HFpEF challenging.

**Figure 1:**
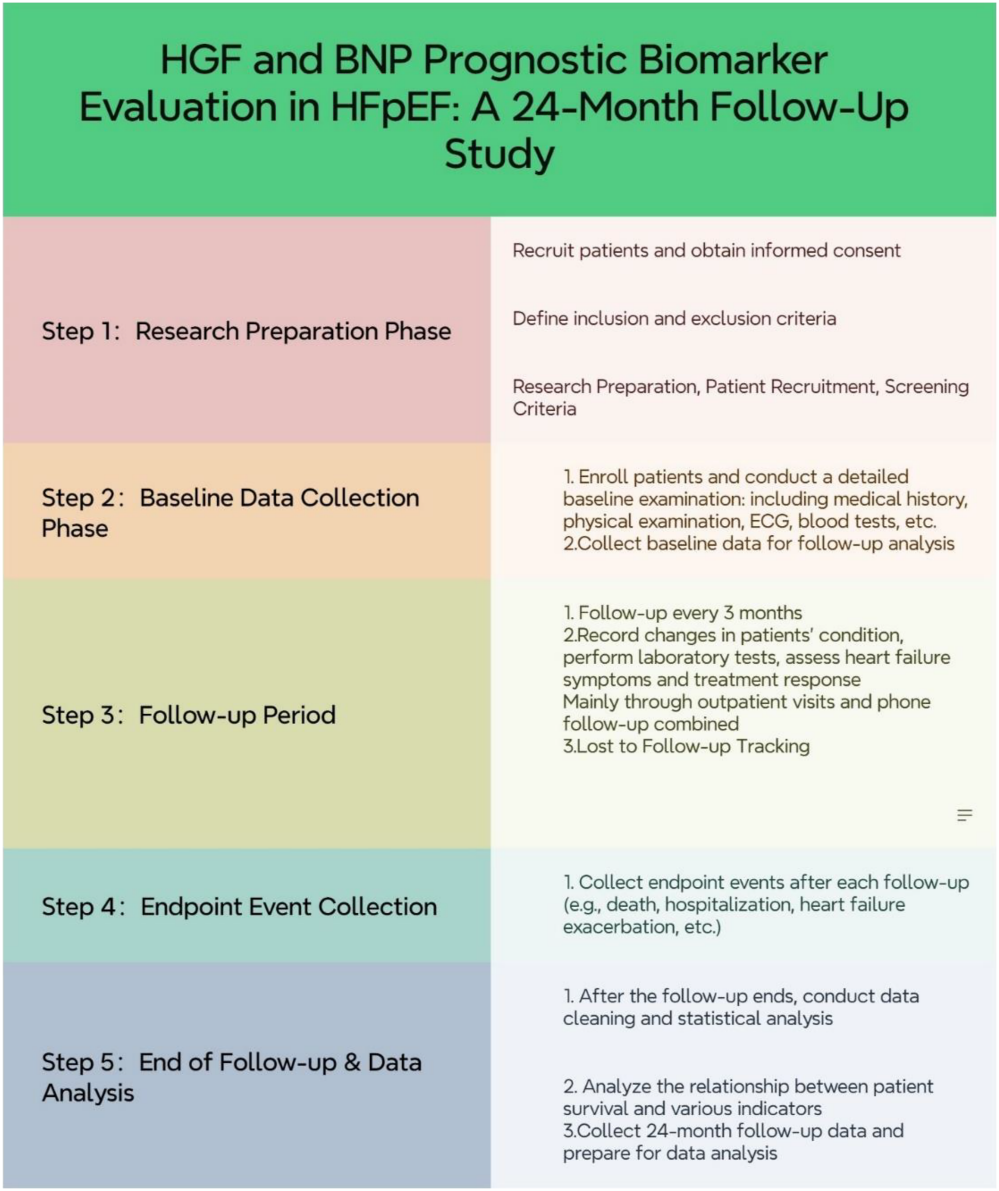
Research process flow chart.

Systemic inflammation has been implicated in the development of HFpEF. Several conditions that are prevalent in HFpEF patients, such as overweight/obesity (especially pericardial fat), hypertension, diabetes, and chronic obstructive pulmonary disease, can trigger systemic inflammatory response. This in turn can cause ventricular remodeling and diastolic dysfunction through various signaling pathways (2–4).Other mechanisms that may contribute to the pathogenesis of HFpEF include adiponectin deficiency and endothelial dysfunction (5, 6). These factors can lead to adverse cardiac and vascular changes, such as left ventricular hypertrophy, concentric remodeling, reduced longitudinal systolic function, right ventricular dysfunction, pulmonary hypertension, vascular stiffness and dysfunction, and cardiac dyssynchrony (7).These alterations can result in elevated left ventricular end-diastolic pressure and clinical signs and symptoms of HFpEF.

Hepatocyte growth factor (HGF) is a mesenchymal cell-derived factor that plays an essential role in the embryonic development of epithelial and endothelial cell lines. HGF has anti-inflammatory, anti-apoptotic, anti-fibrotic, and pro-angiogenic effects that may be beneficial for tissue repair and regeneration (8). However, epidemiological studies have shown that elevated HGF levels are associated with increased risk of coronary heart disease, stroke, peripheral arterial disease, and HFpEF (9–13). Furthermore, the Multi-Ethnic Study of Atherosclerosis (MESA) demonstrated that higher levels of HGF were independently linked to left ventricular hypertrophy, concentric remodeling, and decreased left ventricular end-diastolic volume after a 10-year follow-up in participants from six US communities (14). B-type natriuretic peptide (BNP) has become a potential biomarker for HFrEF prognosis, but there are few studies on the prognosis of HFpEF.

There is a lack of studies evaluating the association between HGF and BNP and subsequent all-cause mortality outcomes in HFpEF patients. Therefore, this study aimed to assess the association between HGF and BNP and all-cause mortality in patients with HFpEF.

## 2 Materials and methods

### 2.1 Study population

This study enrolled patients with HFpEF who were hospitalized in the Department of Cardiology of the First Affiliated Hospital of Anhui Medical University between November 2020 and November 2021.

The inclusion criteria were as follows: patients with HF symptoms [classified as class II to IV by the New York Heart Association (NYHA)], left ventricular ejection fraction (LVEF) of 50% or higher, age of 18 years or older, and increased levels of natriuretic peptides (BNP] of 35 pg/ml or more, or N-terminal pro-B-type natriuretic peptide [NT-proBNP] of 125 pg/ml or more for patients with sinus rhythm; or BNP of 105 pg/ml or more, or NT-proBNP of 365 pg/ml or more for patients with atrial fibrillation).The exclusion criteria were as follows: acute coronary syndrome, severe systemic diseases (such as rheumatic immunological diseases or malignant tumors), expected life span <3 years, severe chronic pulmonary diseases requiring home oxygen therapy, and contraindications to optimal drug therapy according to the 2016 European Society of Cardiology guidelines for HF (15). All patients provided informed consent in accordance with the Declaration of Helsinki, and the Ethics Committee of the First Affiliated Hospital of Anhui Medical University approved this study. The following flowchart illustrates the comprehensive process of study design, patient enrollment, follow-up, and data collection in this research (see Figure 1 for details).

### 2.2 Analytical parameters

The medical history of the enrollees was collected and a comprehensive physical examination was performed. The following data were recorded: gender, age, body mass index (BMI), heart rate, blood pressure, comorbidities, prescription drugs, NYHA functional classification, and echocardiographic evaluation results. Peripheral blood was drawn within 24 h of admission and the complete blood cell count and other laboratory parameters were analyzed, including HGF, BNP, creatinine, aspartate aminotransferase (AST), alanine aminotransferase (ALT), electrolytes (sodium and potassium), and high-sensitivity C-reactive protein (hsCRP). The estimated glomerular filtration rate (eGFR) was calculated using the measured blood creatinine level (16). The plasma HGF levels were measured by enzyme-linked immunosorbent assay method, and the left ventricular ejection fraction was calculated by Simpson method using echocardiography. The main endpoint of this study was all-cause mortality during 24 months of follow-up, which was obtained mainly through outpatient visits, hospital records, or telephone follow-up. All patients were followed up once every 3 months.

### 2.3 Statistical analysis

The data were analyzed using SPSS 20.0 software. The mean ± standard deviation was reported for continuous variables with normal distribution, and the two independent samples *T*-test was performed for comparison. For multiple group comparison, the non-parametric test was applied (Kruskal–Wallis test for 3 groups, Mann–Whitney *U*-test for 2 groups). Continuous measurement data without normal distribution were presented as M (Q1, Q3) and compared by the Kruskal–Wallis *H*-test. The count data were expressed as percentages and examined by the chi-square test. The patients were stratified into two groups based on their survival status. The independent risk factors for all-cause mortality in HFpEF were identified by multivariate logistic regression analysis. The ROC curve analysis was conducted to determine the best cut-off value of HGF protein concentration for predicting all-cause mortality in HFpEF, and the patients were classified according to this value. The Kaplan–Meier analysis was used to explore the association between HFpEF subgroups and death time. A *p*-value of less than 0.05 was considered statistically significant.

## 3 Results

This study included 460 HFpEF patients, with 48 lost to follow-up, yielding a 24-month follow-up completion rate of 89.57%. The 10.43% loss-to-follow-up was primarily attributed to COVID-19 lockdowns restricting access to outpatient care (32 cases), psychological concerns such as fear of infection (10 cases), and logistical challenges due to patients' reliance on busy family members (6 cases).

Among 412 patients with 24 months of complete follow-up data, the most common comorbidities were hypertension (63.59%), coronary heart disease (50.48%), atrial fibrillation (37.14%), ischemic stroke (21.84%), and diabetes (20.87%). In the study population, 223 patients presented with NYHA II (54.13%), 143 patients presented with NYHA III (34.71%), and 46 patients presented with NYHA IV (11.16%). Baseline characteristics are shown in Table 1.

**Table 1:** Baseline data of HFpEF patients at the time of inclusion were compared between the survival group and the death group.

Parameter	Survival group (*n* = 330)	Death group (*n* = 82)	All patients (*n* = 412)
Age (years)	71.57 ± 11.24*	75.43 ± 10.467	72.34 ± 11.18
Males, *n* (%)	183 (55.5%)	43 (52.4%)	226 (54.8%)
SBP (mmHg)	168.38 ± 22.45	172.76 ± 20.46	169.25 ± 22.11
DBP (mmHg)	85.63 ± 23.14*	91.54 ± 13.21	86.81 ± 21.65
BMI (kg/m^2^)	22.38 ± 7.35	20.97 ± 8.96	22.11 ± 7.69
HGF (pg/ml)	1,423.86 (1,189.09, 1,615.16)*	1,599.05 (1,338.56, 1,794.24)	1,455.17 (1,219.27, 1,662.70)
BNP (pg/ml)	446.59 ± 502.33*	880.76 ± 803.811	531.07 ± 597.63
WBC (10^9^/L)	6.70 ± 2.53	6.740 ± 2.39	6.70 ± 2.50
RBC (10^12^/L)	4.06 (3.61, 4.51)*	3.93 (3.415, 4.35)	4.26 ± 5.38
HGB (g/L)	122.09 ± 22.36*	115.01 ± 24.52	120.70 ± 22.95
PLT (10^9^/L)	179.92 ± 67.92	180.64 ± 77.93	180.06 ± 69.91
TP (g/L)	65.12 ± 6.69*	63.12 ± 7.328	64.73 ± 6.86
ALB (g/L)	38.9 (36.4, 41.83)*	36.6 (34.15, 40.05)	38.6 (35.9, 41.5)
ALT (U/L)	26.79 ± 23.55	35.35 ± 76.60	28.47 ± 40.02
AST (U/L)	35.45 ± 50.21	37.84 ± 61.65	35.92 ± 52.58
UA (umol/L)	374 (302.25, 468.00)*	431 (323.5, 513.5)	380 (306.5, 475.5)
LDH (U/L)	243.60 ± 170.45	245.53 ± 137.95	243.98 ± 164.34
BUN (mmol/L)	9.01 ± 21.144	9.63 ± 7.62	9.13 ± 19.23
CRE (umol/L)	93.10 ± 72.70	106.27 ± 61.29	95.71 ± 70.72
eGFR (ml/min/1.73 m^2^)	77.57 ± 24.37*	65.62 ± 26.53	75.21 ± 25.23
Na^+^ (mmol/L)	138.97 ± 3.56	138.36 ± 3.99	138.85 ± 3.66
K^+^ (mmol/L)	3.87 (3.60, 4.20)*	4.06 (3.64, 4.48)	3.90 (3.61, 4.23)
Cl^+^ (mmol/L)	104.7 (102.15, 107.00)	104.15 (100.82, 107.03)	104.60 (101.80, 107.00)
Ca^+^ (mmol/L)	2.26 (2.17, 2.36)	2.245 (2.13, 2.33)	2.25 (2.17, 2.35)
CK (U/L)	223.52 ± 917.13	109.46 ± 112.55	200.28 ± 820.95
CKMB (U/L)	24.49 ± 73.61	14.64 ± 9.95	22.51 ± 66.06
HbA1c (%)	6.54 ± 1.149	6.81 ± 1.47	6.60 ± 1.23
D-Dimer (ug/ml)	0.98 ± 1.52*	1.77 ± 2.33	1.13 ± 1.74
TSH (mIU/L)	3.69 ± 7.93	3.67 ± 5.11	3.69 ± 7.46
T3 (nmol/L)	1.859 ± 7.99	1.231 ± 0.5057	1.73 ± 7.17
T4 (nmol/L)	98.55 (82.55, 117.6)	95.05 (77.17, 106.5)	97.40 (82.00, 112.80)
TC (mmol/L)	3.91 ± 1.096	3.90 ± 1.14	3.91 ± 1.10
TG (mmol/L)	1.268 ± 0.72	1.29 ± 0.72	1.27 ± 0.72
HDL-C (mmol/L)	1.04 (0.88, 1.22)	1.01 (0.87, 1.15)	1.03 (0.88, 1.22)
LDL-C (mmol/L)	2.312 ± 0.8581	2.405 ± 0.8005	2.33 ± 0.85
VLDL-C (mmol/L)	0.465 ± 0.2616	0.481 ± 0.2682	0.47 ± 0.26
CRP (mg/L)	16.751 ± 29.044	25.585 ± 44.0136	18.68 ± 32.99
Cardiac ultrasound parameters			
AO (cm)	3.2 (3.00, 3.39)	3.22 (2.93, 3.41)	3.20 (2.99, 3.39)
IVSD (cm)	1.12 ± 0.25	1.16 ± 0.30	1.13 ± 0.26
LVD (cm)	4.93 ± 0.63	4.98 ± 0.62	4.94 ± 0.63
SV (ml)	69.19 ± 21.23	70.35 ± 21.56	69.42 ± 21.28
FS (%)	31.61 ± 3.71	31.85 ± 4.70	31.66 ± 3.93
LVPWD (cm)	0.975 ± 0.1308	1.001 ± 0.2006	0.98 ± 0.15
LVEF (%)	58.56 ± 4.927	58.18 ± 4.907	58.49 ± 4.92
QRS wave duration (ms)	104.71 ± 47.208	104.52 ± 39.897	104.67 ± 45.82
Q-T interval	399.02 ± 68.537	392.05 ± 75.46	397.66 ± 69.91
QTc (ms)	440.81 ± 44.418	446.85 ± 45.507	442.00 ± 44.64
Smoking history	65 (19.7%)	18 (22.0%)	83 (20.1%)
MRA	172 (52.1%)	50 (60.9%)	222 (53.9%)
ACEI/ARB/ANRI	191 (57.87%)	40 (48.78%)	231 (56.1%)
SGLT2 inhibitors	40 (12.1%)	13 (15.9%)	53 (12.9%)
Beta-blocker	192 (58.2%)	50 (61.0%)	242 (58.7%)
hypertension	209 (63.3%)	53 (64.6%)	262 (63.6%)
Diabetes mellitus type II	71 (21.5%)	15 (18.3%)	86 (20.9%)
CAD	162 (49.1%)	46 (56.1%)	208 (50.5%)
IS	73 (22.1%)	17 (20.7%)	90 (21.8%)
AF	120 (36.4%)	33 (40.2%)	153 (37.1%)
ICD/CRT-D	14 (4.24%)	4 (4.8%)	18 (4.4%)

SBP, systolic blood pressure; BMI, body mass index; WBC, white blood cells; PLT, platelets; LDH, lactate dehydrogenase; BUN, blood urea nitrogen; CRE, creatinine; CKMB, creatine phosphokinase isoenzyme; HbA1c, glycosylated hemoglobin; TSH, thyrotropin; T3, triiodothyronine; TC, total cholesterol; TG, triglyceride; HDL-C, high density lipoprotein cholesterol; LDL-C, low-density lipoprotein cholesterol; VLDL-C, very low density lipoprotein cholesterol; CRP-C, reactive protein; AO, aortic diameter; IVSD, Interventricular septal thickness; LVD, left interior diameter; SV, stroke volume per minute; FS, left ventricular shortening fraction; LVPWD, left ventricular posterior wall thickness; LVEF, left ventricular ejection fraction; MRA, mineralocorticoid receptor antagonist; ACEI, angiotensin-converting enzyme inhibitors; ARB, angiotensin II receptor blocker; ARNI, angiotensin receptor neprilysin inhibitor; SGLT2 inhibitors, sodium-dependent glucose transporters 2 inhibitors; CAD, coronary artery disease; IS, ischemic stroke; AF, atrial fibrillation; ICD, implantable cardioverter-defibrillator; CRT-D, cardiac resynchronization therapy defibrillator.

* *P* < 0.05, compared with death group.

### 3.1 Basic clinical characteristics of HFpEF patients during 24 months

A total of 412 patients completed 24 months of follow-up, of which 82 (19.90%) had all-cause mortality endpoint events. There was no significant difference between the two groups in blood lipids, glycated hemoglobin, cardiac drug treatment, hypertension, coronary heart disease, history of type 2 diabetes, history of atrial fibrillation, etc. (*P* > 0.05).

The two groups differed significantly in age, diastolic blood pressure (DBP), HGF, BNP, red blood cells (RBC), hemoglobin (HGB), estimated glomerular filtration rate (eGFR), total protein (TP), albumin (ALB), uric acid (UA), potassium (K^+^), D-Dimer, and tetraiodothyronine (T4) (*P* < 0.05). Among them, the survival group had lower age, HGF, BNP, D-Dimer, UA, and K^+^ and higher DBP, RBC, HGB, eGFR, TP, ALB, and T4 than the death group (*P* < 0.05).

### 3.2 Multivariate logistic regression analysis of HFpEF patients during 24 months

After adjusting for covariates including gender, BMI, SBP, DBP, HR, HGB, blood lipids, QRS wave duration, LVEF, left ventricular end-diastolic diameter (LVEDd), anti-heart failure drug use, comorbidities (history of hypertension, diabetes, ischemic stroke, atrial fibrillation), implantable cardioverter-defibrillator (ICD)/cardiac resynchronization therapy (CRT)implantation, multivariate logistic regression analysis of CHF patients after 24 months of follow-up showed that HGF, BNP, TP, eGFR, and T4 were independent risk factors for all-cause mortality in HFpEF patients (*P* < 0.05) (Table 2).

**Table 2:** Multivariate logistic regression analysis of all-cause mortality at 24 months.

Risk factor	*β* value	OR value	95% CI	*P*-value
HGF	0.028	1.028	1.016–1.040	0.000
BNP	0.001	1.001	1.0000–1.001	0.002
TP	−0.044	0.957	0.917–1.000	0.048
eGFR	−0.020	0.980	0.968–0.993	0.002
T4	−0.013	0.988	0.976–1.000	0.042

### 3.3 Comparison of general clinical data and HGF and BNP levels among NYHA functional subgroups

As the NYHA functional grading increased, HGF and BNP levels increased, and there was a significant difference between NYHA IV and NYHA II and NYHA III (*P* < 0.05) (Table 3).

**Table 3:** Comparison of general clinical characteristics and HGF levels among NYHA functional class subgroups based on baseline data at enrollment (¯x ± S).

Group	Number of cases	HGF (pg/mL)	BNP (pg/mL)	TP (g/L)	eGFR (mL/min/1.73m^2^)	T4 (nmol/L)
II level	223	1400.67 ± 283.80	425.88 ± 508.06	65.16 ± 6.84	79.31 ± 23.78	100.59 ± 34.57
III level	143	1424.59 ± 289.78	567.84 ± 620.33	64.378 ± 6.77	70.37 ± 2.15*	97.81 ± 28.17
IV level	46	1576.19 ± 231.57*^,^**	953.59 ± 746.56*^,^**	63.73 ± 7.19	70.35 ± 27.72*	93.81 ± 24.29

* *P* < 0.05, compared with NYHA class II.

** *P* < 0.05, compared with NYHA class III.

### 3.4 Serum HGF, BNP, and eGFR and the prognostic value for HFpEF

**Figure 2:**
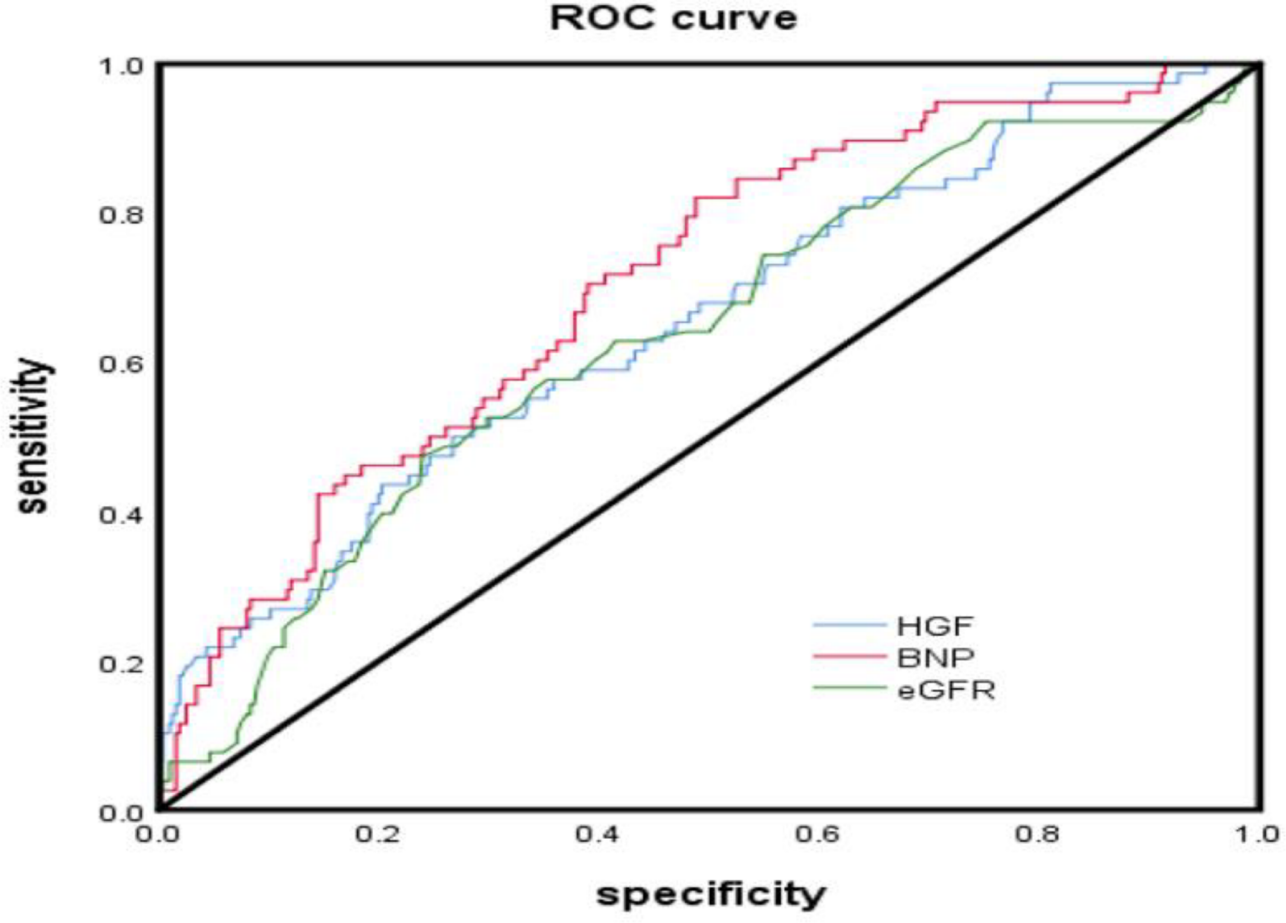
ROC curve analyzed the prognostic value of serum HGF, BNP, eGFR levels in hFpEF after 24 months of follow-up.

According to the collected data, the ROC curve of all-cause mortality was calculated, and the optimal cut-off point of HGF was determined to be 1,598 pg/ml (AUC = 0.645, *P* = 0.000, HR = 3.186 95% CI: 1. 963–5.171), the optimal cut-off point of BNP was 271 pg/ml (AUC = 0.703, *P* < 0.000, HR = 4.494 95% CI: 2.914–6.930), and the optimal cut-off point of eGFR was 114.5 ml/min/1.73 m^2^ (AUC = 0.644, *P* = 0.423) (see Table 4). According to the results of the receiver operating characteristic curve, TP and T4 had *P*-values >0.05, which were not statistically significant, and no further analysis was performed. HGF, BNP, and eGFR had better predictive value for all-cause mortality events in HFpEF during 24 months of follow-up (*P* < 0.05) (Figure 2).

**Table 4:** ROC curve analysis of serum HGF, BNP, eGFR in HFpEF after 24 months of follow-up prognostic value.

Parameter	AUC	95% CI	*P*值
HGF	0.645	0.572–0.718	0.000
BNP	0.703	0.638–0.768	0.000
eGFR	0.644	0.573–0.715	0.000
TP	0.564	0.488–0.640	0.093
T4	0.578	0.508–0.649	0.059

### 3.5 Kaplan–Meier survival curve analysis

**Figure 3:**
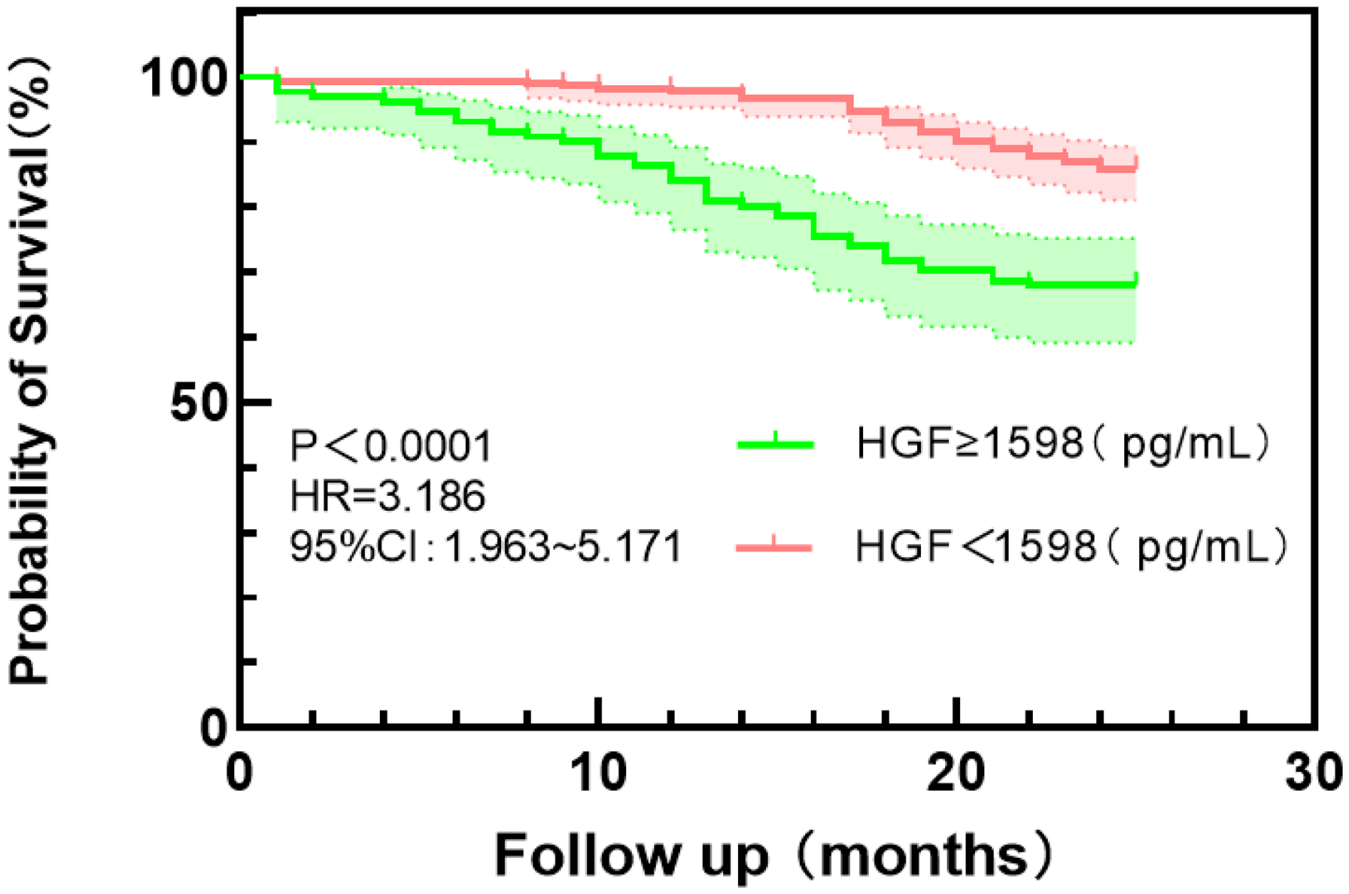
Kaplan–Meier survival curve analysis of HGF grouped by 1,598 pg/ml cut point values.

**Figure 4:**
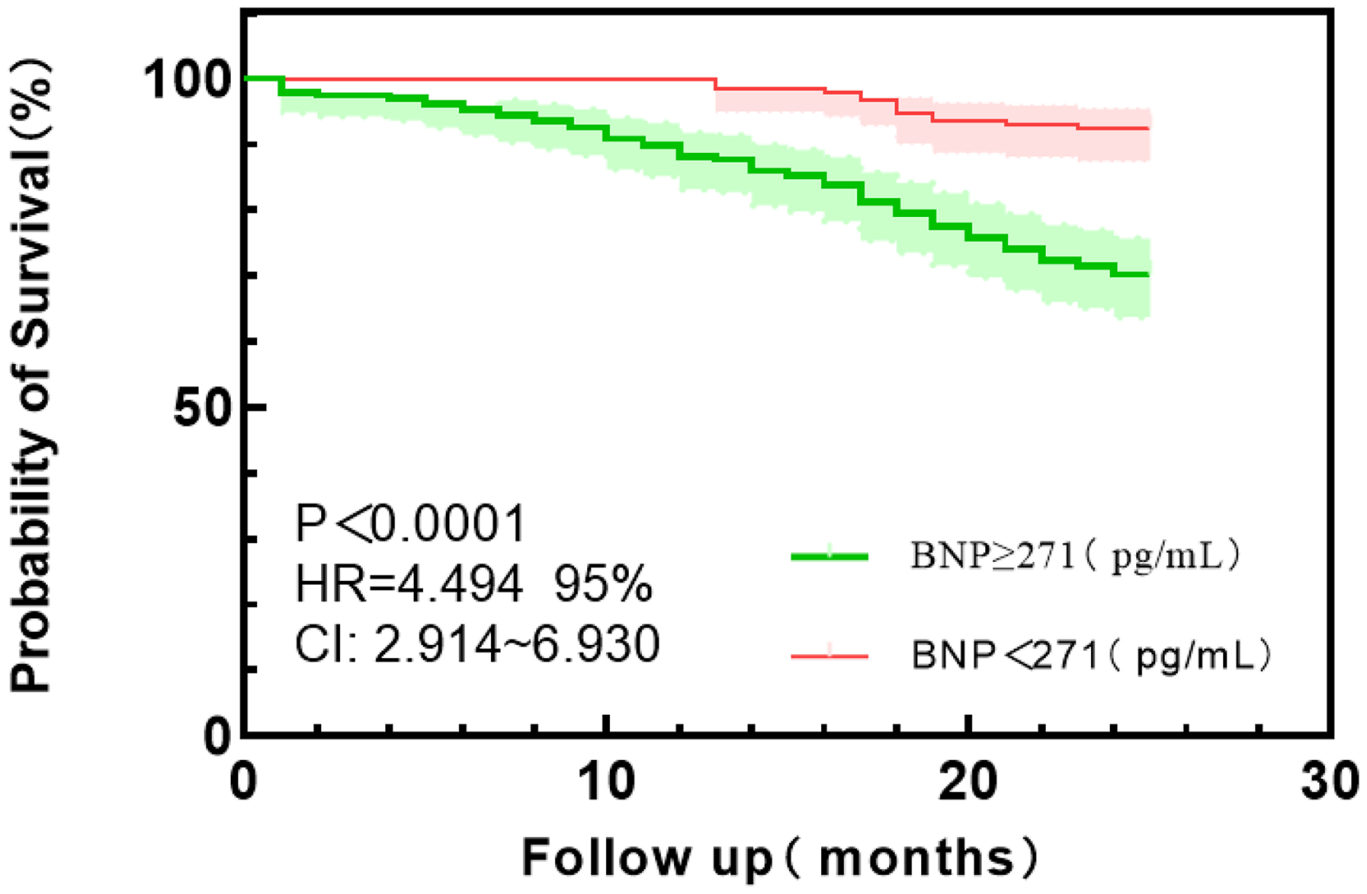
Kaplan–Meier survival curve analysis of BNP grouped by 271 pg/ml cut point values.

**Figure 5:**
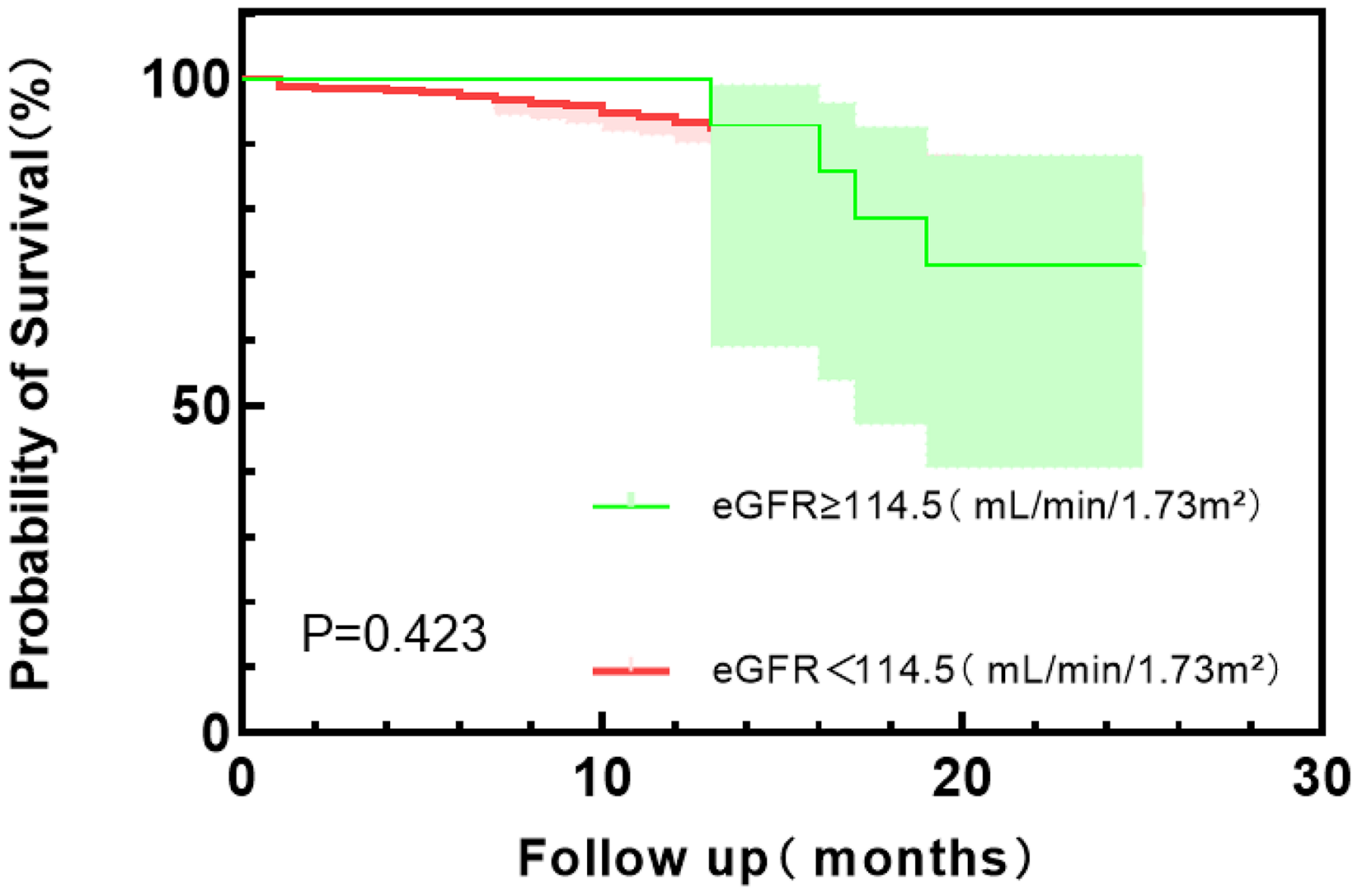
Kaplan–Meier survival curve analysis of eGFR with 114.5 (ml/min/1.73 m^2^) tangential point values.

According to the Youden index, the optimal cut-off values of HGF, BNP, and eGFR were used as the dividing points, and the HFpEF study group was divided into different subgroups. The probability of the main endpoint depended on the plasma concentrations of HGF, BNP, and eGFR, and Kaplan–Meier curve was used for estimation. Kaplan–Meier plot showed that the survival probability of the patients with low HGF and BNP concentrations was significantly higher than that of the patients with high HGF and BNP values (*P* < 0.0001) (see Figure 3 and Figure 4); Kaplan–Meier survival curve analysis showed that there was no significant difference in survival rate between the two groups with eGFR as the cut-off value of 114.5 ml/min/1.73 m^2^ (*P* = 0.423) (Figure 5).

## 4 Discussion

This single-center study enrolled 412 HFpEF patients, of whom 82 (19.90%) had all-cause mortality events within 24 months of follow-up. Multivariate logistic regression analysis showed that HGF, BNP, TP, eGFR, and T4 were independent risk factors for all-cause mortality in HFpEF patients. HGF and BNP levels increased with the increase of NYHA grading level, and there was a significant difference between NYHA IV and NYHA II and NYHA III, indicating that HGF and BNP plasma levels increased significantly when HFpEF patients had more severe symptoms and cardiac decompensation. In the analysis of prognostic indicators for HFpEF, TP and T4 levels had no value for the prognosis of HFpEF, and the increase of HGF and BNP concentrations and the decrease of eGFR level were independent risk factors for all-cause mortality in HFpEF patients. The Kaplan–Meier survival curve analysis of the eGFR group with a cut-off value of 114.5 ml/min/1.73 m^2^ showed that the log-rank test *p*-value was 0.423, indicating that the low eGFR level group did not reduce the risk of all-cause mortality compared with the high level group, and did not increase the survival time. The results of this study suggest that HGF and BNP levels may be prognostic indicators for HFpEF patients, which can help stratify the prognosis of patients.

HFpEF is a prevalent form of heart failure that affects about half of all patients with this condition (17, 18). The pathogenesis of HFpEF is complex and multifactorial, involving aging and the accumulation of major risk factors such as obesity, hypertension, diabetes, and atrial fibrillation. These risk factors can trigger systemic inflammation, natriuretic peptide deficiency, neuroendocrine activation, endothelial dysfunction, autonomic dysfunction, and other mechanisms that impair the diastolic function and compliance of the left ventricle (19); The lack of a clear understanding of the underlying mechanisms of HFpEF leads to poor treatment outcomes and high mortality rates. Cardiovascular death accounts for 60%–70% of all deaths in HFpEF patients (20, 21). Although HFpEF and HFrEF share some common risk factors, they do not necessarily have the same impact on each subtype of heart failure. For instance, obesity is a more prominent risk factor in HFpEF than in HFrEF (22). Moreover, some studies have found that inflammation is more strongly associated with HFpEF than with HFrEF. Therefore, future research should explore the different pathophysiological mechanisms of HFpEF and use blood biomarkers such as inflammation, fibrosis, and others to detect the onset and assess the prognosis of HFpEF.

HGF exhibits potential cardioprotective properties, such as anti-inflammation, anti-apoptosis, anti-fibrosis, and pro-angiogenesis. HGF preserves cardiac function, attenuates fibrosis and infarct size, promotes angiogenesis and cardiomyocyte survival, and stimulates PI3-kinase/Akt pathway in mice with myocardial infarction (23). In a mouse model of myocardial infarction, HGF-treated mice had thicker left ventricular wall in the late infarct area, suggesting that HGF modulates left ventricular remodeling (24). HGF correlates with impaired functional capacity and quality of life in HFpEF patients1 (25). This implies that high HGF levels may indicate the decompensation of the body in heart failure. This is consistent with our finding that the patients who died during the 24-month follow-up had significantly higher HGF levels [1,599.05 (1,338.56, 1,794.24)] than the patients who survived [1,423.86 (1,189.09, 1,615.16)], and the difference was statistically significant (*P* < 0.05). The latest data from cardiology oncology show that HGF is a biomarker associated with the prognosis of cardiac amyloidosis. HGF is significantly elevated in cardiac amyloidosis patients (*P* < 0.001), and HGF level of 205 pg/ml can distinguish cardiac amyloidosis, symptomatic heart failure with left ventricular hypertrophy, and HFrEF patients, with a sensitivity of 86%, specificity of 84%, and area under the curve of 0.88 (95% CI 0.83–0.94). In amyloidosis patients, elevated HGF levels were associated with worse survival rate at median follow-up of 2.6 years, and had higher prognostic accuracy than NT-proBNP and troponin-T (*P* < 0.001) (26).

Cardiac amyloidosis is also one of the causes of HFpEF related to cardiomyopathy. In our study, we can see that HGF is a risk factor for 2-year all-cause mortality in HFpEF, and the optimal cut-off value is 1,598 pg/ml, which is significantly higher than 205 pg/ml. The reason for this is that all the enrolled patients in our study are HFpEF, and we did not distinguish the subgroups of the enrolled patients according to the etiology. Kathleen W. Zhang et al. (26) enrolled 188 patients, divided into 4 groups: cardiac amyloidosis group (*n* = 72), non-cardiac involvement amyloidosis group (*n* = 30), symptomatic heart failure with left ventricular hypertrophy (LVH) group (*n* = 44), and HFrEF group (*n* = 42). The study population was different, so the diagnostic cut-off value was different. Secondly, the follow-up time of this study was longer than our own study (2.6 years vs. 2 years), but the follow-up results were the same: the higher the HGF level, the lower the survival rate.

In another study, 136 CHF patients underwent CRT implantation and their HGF levels were measured before and 3 months after the procedure. The mean HGF level decreased from 1,379 [1,029–1,863] pg/ml to 1,083 [862–1,328] pg/ml after CRT treatment. The main endpoint of the study was 5-year all-cause mortality, which was assessed by multivariate analysis. The results showed that only HGF elevation was an independent predictor of 5-year all-cause mortality (HR = 1.35; 95% CI 1.11–1.64; *P* = 0.003). Therefore, HGF not only predicted the responsiveness of CRT but also the long-term mortality of chronic decompensated HF patients (27). At the same time, it was observed that HGF was significantly reduced to 1,083 pg/ml in patients with heart failure treated by CRT implantation, which was lower than the optimal cutoff value of 1,598 pg/ml for predicting 2-year all-cause mortality in HFpEF patients in our study, which indicates that whether in HFrEF decompensated patients or in HFpEF patients, HGF elevation is an independent predictor of all-cause mortality in two different subgroups of HF. The increase of HGF level may reflect the failure of the cardiovascular system protection pathway, thus identifying the patients with the most unstable clinical condition and the highest risk (24, 28).

MESA study is a cohort study that enrolled 6,597 multi-ethnic patients with atherosclerosis, with a mean age of 62 ± 10 years. The median HGF level was 950 pg/ml, and the study results indicated that HGF was independently associated with HF events, and in the subtype assessment, HGF was significantly associated with recurrent HF events in HFpEF patients, but not with HFrEF (13). Our study enrolled 412 HFpEF patients, and is one of the few studies that evaluated the prognostic value of HGF for HFpEF patients. The endpoint was assessed as death due to all causes, and the ROC curve results showed that HGF could predict the 24-month all-cause mortality risk of HFpEF (AUC = 0.645, *P* = 0.000, HR = 3.186 95% CI: 1.963–5.171). This result is similar to that of Richter et al., who found that HGF was the strongest predictor of cardiovascular mortality in late-stage HF patients (29). But the two studies enrolled different populations, Richter et al. enrolled patients with late-stage systolic heart failure: (a) currently hospitalized for cardiac decompensation, (b) NYHA III or IV at admission, and (c) left ventricular ejection fraction (LVEF) <40% and/or cardiothoracic ratio >0.5.

When HF occurs, left ventricular wall stress increases, myocardial cells stretch, and a large amount of BNP is secreted by myocardial cells. BNP level has a good correlation with left ventricular end-diastolic pressure. Although some studies have suggested that BNP levels are lower in HFpEF than in HFrEF patients with acute decompensated heart failure, serum BNP levels have predictive value for prognosis in both types of heart failure (30). In our study, BNP was significantly higher in the death group than in the survival group in HFpEF patients, and combined with NYHA grading, BNP was significantly higher in NYHA IV than in NYHA II and NYHA III in HFpEF patients, indicating that BNP could reflect the symptoms of cardiac decompensation in this type of patients. Further combined with multivariate logistic regression analysis and ROC curve analysis results, BNP was a risk factor for all-cause mortality events in HFpEF patients, and had a certain predictive value for all-cause mortality events. When BNP was grouped with a cut-off value of 271 pg/ml, there was a statistical difference in the 24-month all-cause mortality rate between the high and low groups (*P* < 0.05).

This study has the following limitations: This study is a single-center, retrospective study, and missing or inaccurate clinical data of patients may interfere with the results. The limited sample size of this study, the relatively short follow-up period, and the absence of advanced diagnostic techniques such as cardiac magnetic resonance imaging (CMR) may introduce potential biases to the study's findings. The accuracy of echocardiography physicians in measuring diastolic dysfunction needs to be further improved, and right heart catheterization can be performed if necessary to identify some potential diastolic dysfunction patients. The ROC analysis does not account for time-to-event data, which limits its applicability in survival analysis. ROC curves are primarily used to assess the accuracy of classification prediction models, particularly in determining optimal cut-off values. However, ROC analysis does not consider time factors, whereas Cox regression may be more suitable for handling survival-related outcomes. Therefore, while the AUC value provides valuable information, it is not a perfect metric and should be interpreted with caution. Given the limited effective treatments available for HFpEF at present, it is crucial to develop methods for early risk stratification and prognostic assessment in HFpEF patients. In future studies, more prospective, well-designed, large-sample, intervention-strategy, and long-term follow-up studies are needed to verify the current conclusions.

## Data Availability

The original contributions presented in the study are included in the article/Supplementary Material, further inquiries can be directed to the corresponding author.
