# Supplementary figures and images for: Hepatocyte growth factor and B-type natriuretic peptide as independent predictors of mortality in HFpEF patients
Source: Front Cardiovasc Med. 2025 Feb 18;12:1512411. doi: 10.3389/fcvm.2025.1512411 (PMC11876389; doi:10.3389/fcvm.2025.1512411)

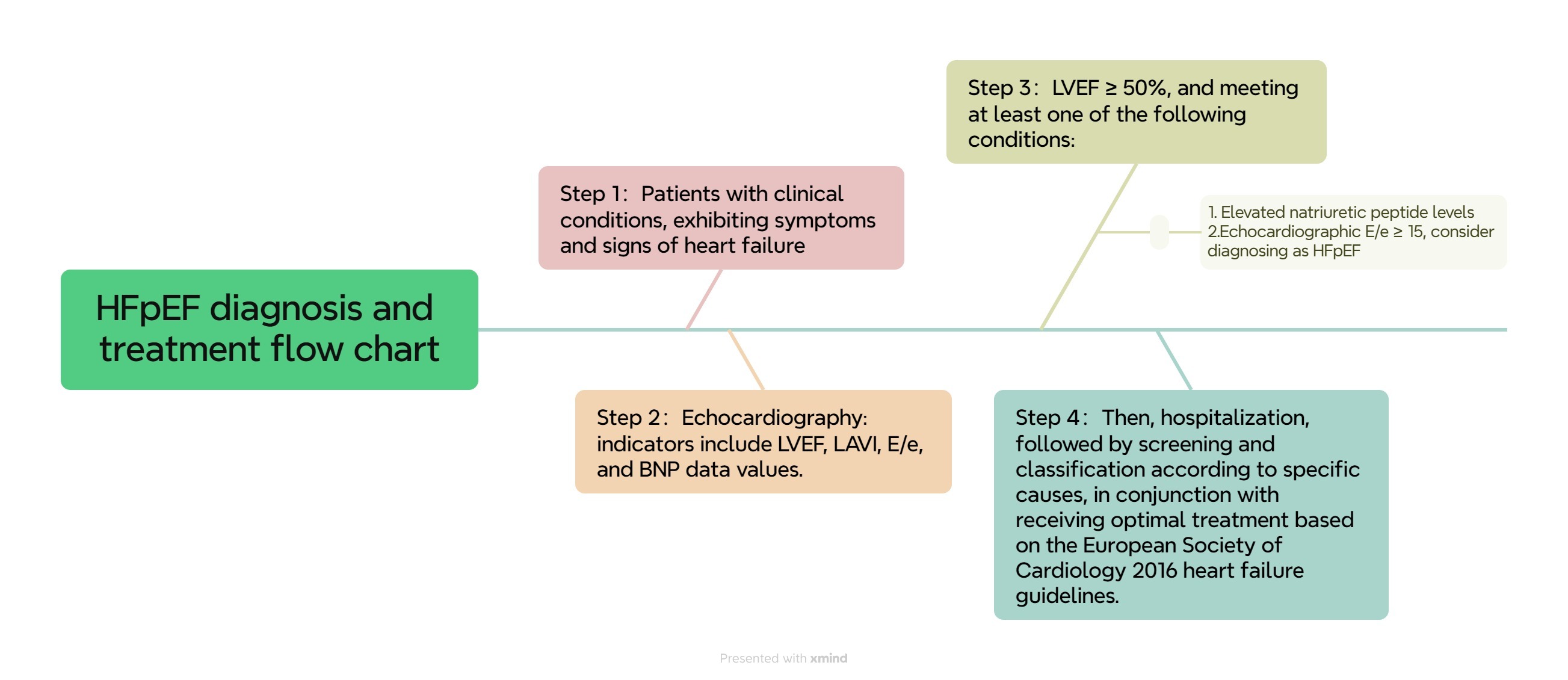

Supplement: Supplementary file 1 [file Image1.jpeg]

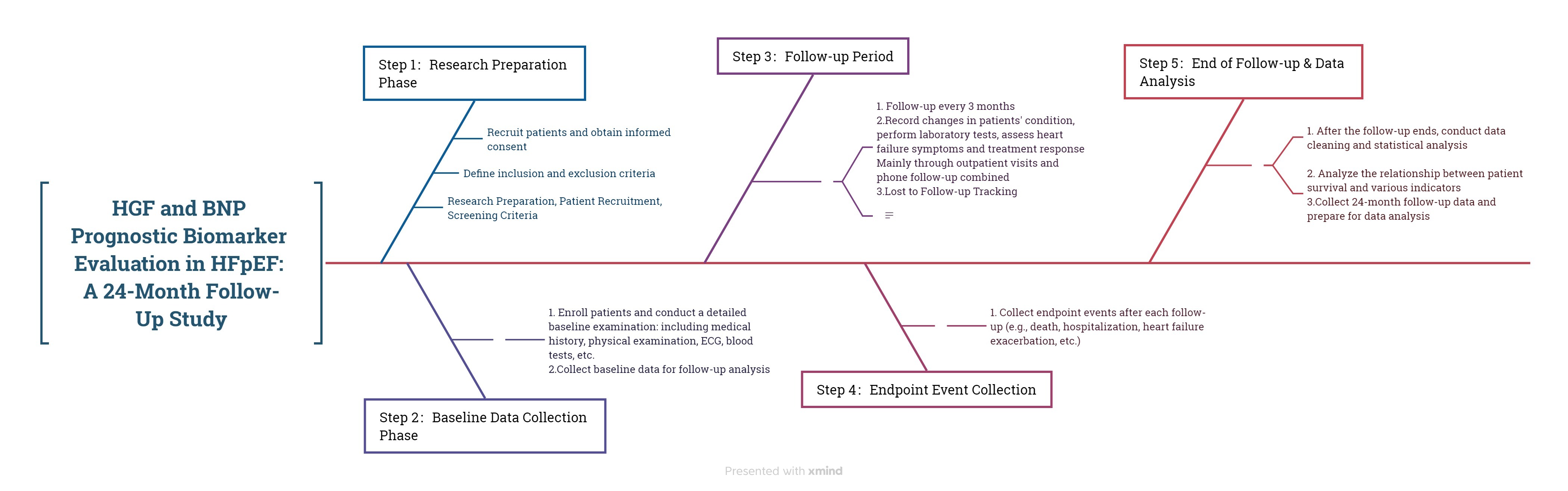

Supplement: Supplementary file 2 [file Image2.jpeg]
